# A new short version of the Posttraumatic Diagnostic Scale: validity among Japanese adults with and without PTSD

**DOI:** 10.1080/20008198.2017.1364119

**Published:** 2017-09-05

**Authors:** Mariko Itoh, Yuri Ujiie, Nobukazu Nagae, Madoka Niwa, Toshiko Kamo, Mingming Lin, Sayuri Hirohata, Yoshiharu Kim

**Affiliations:** ^a^ Department of Adult Mental Health, National Institute of Mental Health, National Center of Neurology and Psychiatry, Tokyo, Japan; ^b^ Tokyo Women’s Medical University, Tokyo, Japan; ^c^ Faculty of Humanities, Fukuoka University, Fukuoka, Japan; ^d^ Wakamatsu-cho Mental and Skin Clinic, Tokyo, Japan; ^e^ Mitsukaidokosei Hospital, Ibaraki, Japan

**Keywords:** Posttraumatic stress disorder, screening scale, validation, Posttraumatic Diagnostic Scale, psychometric properties, re-experiencing symptom

## Abstract

**Background**: Identifying high-risk groups for posttraumatic stress disorder (PTSD) during evacuation situations requires a valid short screening tool. The re-experiencing symptoms of PTSD are considered helpful for distinguishing those with PTSD from those without, as they are thought to be specific to PTSD, have less ambiguity for respondents, and are representative of all PTSD symptoms.

**Objective**: To develop a new short version of the Posttraumatic Diagnostic Scale (PDS) comprising only re-experiencing symptom items.

**Method**: We used existing data (*N =* 169) from our previous study on the Japanese version of the PDS and the Clinician-Administered PTSD Scale (CAPS). The sample included both clinical outpatients (*n =* 106) and university students (*n =* 63), all of whom reported one or more traumatic experiences. We created candidate 2- and 3-item versions of the PDS and compared their psychometric characteristics against the CAPS.

**Results**: The best candidate (comprising items for ‘intrusive images’, ‘nightmares’, and ‘physiological reactions when reminded of the trauma’) demonstrated an area under the curve of .95, 94.8% sensitivity, 86.1% specificity for the best cut-off score of three. The candidate scale also showed a strong correlation with CAPS-evaluated severity score and internal consistency.

**Conclusions**: The brief re-experiencing PDS had good psychometric properties among Japanese adults with and without PTSD.

## Abstract (zh)

题目：新的简版创伤后诊断量表：在患有和未患有PTSD的日本成年群体中的验证

背景：在疏散情景下识别高风险的创伤后应激障碍（PTSD）群体需要一个有效简短的筛选工具。PTSD的再体验症状具有PTSD特异性、对应答者具有较少歧义、对所有PTSD症状有代表性，因此被认为有助于区分患有PTSD和未患有的人群。

目标：开发一个只包含再体验症状题目的新的简版创伤后诊断量表（PDS）。

方法：我们使用之前关于日本版PDS和临床诊断PTSD量表（CAPS）的研究中的现有数据（N=169）。样本中既有临床门诊病人（n=106）也有大学学生(n=69)，他们都报告了一个或以上创伤性经历。我们开发了2个题目和3个题目的备选PDS，并将它们的心理测量特性和CAPS进行对比。

结果：最好的备选量表（包括“闯入性图像”,”噩梦”,”被提醒创伤时的生理反应”）显示出了.95的曲线下面积（area under the curve），最好的划界分（3分）具有94.8%的敏感性和86.1%的特异性。备选量表也表现出了和CAPS症状验证分数的强相关，以及内部一致性。

结论：简版在体验PDS在患有和未患有PTSD的日本成年群体中有很好的心理测量特性。

关键词：创伤后应激障碍，筛选量表，验证，创伤后诊断量表，心理测量特性，再体验症状

## 1. Introduction

Posttraumatic stress symptoms are among the most devastating psychological reactions following life-threatening experiences. However, they tend to be under-diagnosed, particularly during extreme circumstances such as natural and manmade disasters, including earthquakes, terrorist attacks, war crimes, or accidents, when resources for evaluation are typically limited (Disaster Psychiatry Outreach, 2008; Katz, 2011). Despite a general tendency for spontaneous recovery, certain individuals will follow the more chronic path and ultimately develop posttraumatic stress disorder (PTSD). An early evaluation and diagnosis are crucial (Galea, Nandi, & Vlahov, 2005; Watson, Gibson, & Ruzek, 2014).

There is some support for the utility of short PTSD scales (Spoont et al., 2013), such as Breslau’s 7-item scale (Breslau, Peterson, Kessler, & Schultz, 1999), a 4-item scale called the Startle, Physiological arousal, Anger, and Numbness scale (Meltzer-Brody, Churchill, & Davidson, 1999), and a 4-item scale called the Primary Care PTSD Screen (PC-PTSD) (Prins et al., 2003), as well as longer scales such as the PTSD Checklist – Civilian version (PCL) (Blanchard, Jones-Alexander, Buckley, & Forneris, 1996) or Posttraumatic Diagnostic Scale (PDS) (Foa, 1995; Foa, Cashman, Jaycox, & Perry, 1997). For example, the PC-PTSD has shown sufficient validity among veterans (Calhoun et al., 2010; Prins et al., 2003), active-duty soldiers (Bliese et al., 2008), and patients (Freedy et al., 2010; Kimerling, Trafton, & Nguyen, 2006) when using the structured clinical interview as the gold standard (Bliese et al., 2008; Calhoun et al., 2010; Kimerling et al., 2006; Prins et al., 2003). These short scales offer the advantages of saving time and resources, which are especially necessary during the flood of support, activities, documentation, and assessments that occurs in the aftermath of a disaster or accident compared to scales with more than 20 items.

Considering that short scales must consist entirely of clear and essential questions for screening people during an evacuation situation, we assume that items on re-experiencing symptoms could be useful for the following three reasons. First, re-experiencing symptoms are more specific to PTSD compared to hyperarousal or numbing symptoms. Hyperarousal, despite being a main symptom of PTSD, are often observed in other disorders (e.g. generalized anxiety disorder, caffeine intoxication, tobacco withdrawal). Similarly, numbing symptoms are often confused with negative symptoms of schizophrenia, as well as anhedonia in depression (Pandya, 2011).

Second, questions regarding hyperarousal and avoidance symptoms tend to be more ambiguous for evacuation survivors, limiting their utility. For example, the questions concerning ‘hyper-alert’ or ‘trouble falling asleep’ (hyperarousal symptoms) could be easily confused with the anxiety resulting from being in an unfamiliar environment or real-life problems after the event (e.g. aftershocks of an earthquake, problematic behaviours of the abusers or other family members). In addition, items such as ‘trying to avoid activities, situations, or places that remind you of the trauma’ or ‘psychological isolation’ (an avoidance symptom) might also be ambiguous for people during an evacuation situation. These items could be endorsed ‘almost all’ of the time because evacuees are separated from a familiar place/people. Conversely, respondents could respond ‘not at all’ because once they have already evacuated, avoidance was no longer needed, or there is a need to communicate with other evacuees or supporters to receive help or information. Furthermore, certain avoidance items may be more difficult to answer, especially for people deprived of sufficient time or cognitive resources immediately following a trauma. That is, items such as ‘trying to avoid thoughts or feelings related to the trauma’, ‘trying to avoid activities, situations, or places that remind you of the trauma’, or ‘not being able to remember important parts of the trauma’ requires respondents to count the ‘absence’ of an action (not doing something). This may be more difficult than counting the ‘existence’ of action (frequency of doing something, e.g. intrusive images). For these reasons, there are doubts surrounding the inclusion of hyperarousal or avoidance symptoms within a self-administered screening scale for survivors of an evacuation scenario.

Third, items on re-experiencing symptoms may be representative of overall severity of PTSD symptoms. Lang and Stein (2005) have shown that the re-experiencing items of the 17-item PCL (Blanchard et al., 1996; Lang & Stein, 2005; Tiet, Schutte, & Leyva, 2013) had the strongest correlations with total PTSD severity score. Furthermore, an abbreviated version of the PCL scale comprising only the two re-experiencing items had good validity for a diagnosis via the PTSD section of the Composite International Diagnostic Interview Version 2.1 (a sensitivity of .96 and a specificity of .58). Similarly, a 2-item re-experiencing PCL scale had good validity comparable to the PC-PTSD according to the areas under the curve (AUCs), which are calculated through a receiver-operating characteristic (ROC) analysis; the former demonstrated AUCs of .77–.88 (Lang & Stein, 2005; Tiet et al., 2013) and the latter AUCs of .80–.88 (Bliese et al., 2008; Calhoun et al., 2010; Tiet et al., 2013). In addition, adding avoidance and hyperarousal symptoms to this re-experiencing scale did not always result in increases of the AUCs: the AUCs for the 3-, 4-, and 6-item versions were .86, .86, and .89 (Lang & Stein, 2005); .84, .85, and .84 (in a sample of patients with substance use disorder; Tiet et al., 2013); and .77, .78, and .77 (in a sample of general mental health patients; Tiet et al., 2013), respectively. In other words, the results suggest that even a short scale comprising only two re-experiencing items could have comparable diagnostic validity to 3-, 4-, or 6-item version scales that include items on avoidance and hyperarousal.

The purpose of this study was to develop a short re-experiencing scale from the items of the PDS that corresponds to a PTSD diagnosis made via the Clinician-Administered PTSD Scale (CAPS). Although previous studies developed abbreviated scales comprising items that were highly correlated with a PTSD severity score, they did not confirm whether these scales were optimal for determining a PTSD diagnosis made via the CAPS. Thus, in this study, we created short scales by systematically choosing a set of items with the highest validity for a PTSD diagnosis and sufficient internal consistency.^1^ While PTSD severity is generally related to PTSD diagnosis, there could be symptoms that contribute substantially to the severity score but are less relevant for an accurate diagnosis (e.g. A2 criterion, which was deleted in the DSM-5; Osei-Bonsu et al., 2012). We compared 2- and 3-item versions of the new re-experiencing scales, because a screening scale can never be too short in terms of reducing the burden on respondents, but longer scales can increase the likelihood of correctly identifying the concept of concern. We used the PDS to develop a short scale because it has as high validity as other self-rating scales such as the PCL (Adkins, Weathers, McDevitt-Murphy, & Daniels, 2008), corresponds to the DSM criteria for PTSD, and has been validated in Japanese (Itoh et al., 2017).

## 2. Methods

### 2.1. Participants

We used data from the PDS and CAPS collected in our previous study (Itoh et al., 2017) with clinical and non-clinical samples (*N =* 225). The data were screened and excluded if (1) the complete item data were not accessible (*n =* 3) or (2) there was no listed traumatic event in the checklist on Part 1 of the PDS (*n =* 53, nearly half of the non-clinical sample). In the latter case, further evaluation for PTSD symptoms was stopped because the respondents had no trauma-related symptoms. If one or more traumas were checked on Part 1 of the PDS, the respondents proceeded to the following questions that assessed symptoms. The index trauma reported on the PDS was the same as obtained from the CAPS interview. We consequently analysed data from a total of 169 participants; 106 were outpatients who visited the psychiatric ward for trauma therapy at a women’s clinic at a medical university located in Tokyo, Japan, and the remaining 63 were undergraduates who reported one or more subjective traumatic experiences and consented to participate in a study organized by their university. Ethical clearance was obtained from the ethics committees of our affiliated universities.

### 2.2. Instruments

#### 2.2.1. Posttraumatic Diagnostic Scale (PDS/DSM-IV)

In the present study, responses to the full-version of the PDS were used to examine the short scales. For example, we chose responses to re-experiencing symptoms of B1 and B2 from the five re-experiencing items for a 2-item candidate scale. In total, we created 10 patterns of 2-item candidate scales and another 10 patterns of 3-item candidate scales.

The PDS (Foa, 1995; Foa et al., 1997) is a self-rating inventory that corresponds to the diagnostic criteria for adult PTSD of the DSM-IV. The Japanese version (Itoh et al., 2017) was used in this study; its validity is high (AUC of .97 against the CAPS diagnosis, the sensitivity of 97.0%, and specificity of 93.6%). The PDS comprises four parts assessing traumatic experiences (Part 1 and 2), symptom severity over the past month corresponding to Criteria B–D of the DSM-IV (Part 3), and functional disorder (Part 4).

The responses to Part 3 of participants who reported having one or more trauma in Part 1 were used in the present study. We analysed the 4-point scale responses (0 = ‘not at all or only one time’ to 3 = ‘five or more times a week/almost always’) to the five re-experiencing symptoms (Criterion B). These symptoms were (B1) intrusive images, (B2) nightmares, (B3) reliving of the trauma, (B4) emotionally upset when reminded of the trauma, and (B5) physical reactions when reminded of the trauma (Table 1).

**Table 1.** Proportion of participants who answered the re-experiencing symptoms among Japanese participants with and without PTSD.

			CAPS (DSM-IV)	
			PTSD+ *n =* 97	PTSD- *n =* 72
Criteria in DSM-IV	Re-experiencing symptoms of PTSD	Overall *N =* 169	% in each group	
B1	Intrusive images	119	96.91	34.72
B2	Nightmares	92	88.66	8.33
B3	Reliving of the trauma	88	79.38	15.28
B4	Emotionally upset when reminded of the trauma	131	98.97	48.61
B5	Physiological reactions when reminded of the trauma	113	96.91	26.39

PTSD, posttraumatic stress disorder; DSM-IV, Diagnostic and Statistical Manual of Mental Disorders, 4th ed.; CAPS, Clinician-Administered PTSD Scale. PTSD+ indicates that participants met CAPS-diagnosed PTSD criteria.

#### 2.2.2. Clinician-Administered PTSD Scale (CAPS/DSM-IV)

The CAPS (Blake et al., 1995) is a semi-structured interview known as the gold standard for diagnosing and assessing the symptom severity of PTSD. We used the Japanese version of the CAPS (Asukai, Hirohata, Kato, & Konishi, 2003) as the external criterion for evaluating the validity of the 2- or 3-item versions of the PDS. The CAPS was designed to assess the frequency (0 = ‘never’ to 4 = ‘daily or almost every day’) and intensity (0 = ‘none’ to 4 = ‘extreme’) of each of the 17 DSM-IV symptoms and associated features of PTSD. Blake et al. (1995) recommended that a frequency score of ‘1’ and an intensity score of ‘2’ are required for a particular symptom to meet the DSM-IV diagnostic criteria for PTSD; thus, we followed this recommendation for the present study. A severity score was calculated by summing the frequency and intensity scores (range: 0–136). All of the CAPS interviewers (clinical psychologists or graduate students majoring in clinical psychology) had received specialist training on administration of the CAPS.

### 2.3. Data analysis

To obtain a detailed description of the performance of the 2- or 3-item candidate scales in diagnosing PTSD, we compared the AUCs of each candidate. The AUCs were calculated for the total score of each 2- or 3-item candidate scale (range: 0–6 for the 2-item scales and 0–9 for the 3-item scales) against the CAPS diagnostic outcome (i.e. a PTSD diagnosis of ‘positive’ or ‘negative’). We further calculated the sensitivity and specificity at the optimal cut-off score (where the sum of the sensitivity and specificity was maximal). In addition, to evaluate the validity of the candidate scales in assessing PTSD symptom severity, we calculated Pearson’s correlation coefficients between the total score of each candidate scale and the CAPS symptom severity score. The internal consistency of the candidate scales was also examined using Cronbach’s alpha coefficients.

All of the analyses were conducted with the entire sample rather than separately for the clinical and non-clinical subsamples. Ideally, it would be desirable to choose a sample within the spectrum of exceedingly mild to severe symptoms in order to examine the validity of this screening tool, but our available data mostly comprised people with severe symptoms (i.e. a clinical subsample) and people with mild symptoms (i.e. non-clinical subsample). In such subsamples, assuming that all of the participants in the clinical subsample are truly positive for PTSD, the sensitivity and specificity of a scale would be 100% and 0%, if the scale has perfect validity. Similarly, assuming that all of the participants in the non-clinical subsample are truly negative for PTSD, the sensitivity and specificity of a scale would be 0% and 100%, if the scale has perfect validity. Thus, separate analyses for each subsample would not provide meaningful results for evaluation and, in the present study, the analysis was conducted on the combined sample to include a more diverse range of people.

All analyses were performed with SPSS Statistics 23 or the R version 3.2.4 Revised Epi package (plots of the ROC and calculation of the optimal cut-off scores; there were no differences between both software programs). All parameters were calculated with a 95% confidence interval.

## 3. Results

### 3.1. Characteristics of the study participants

The characteristics of the clinical and non-clinical subsamples are shown in Table 2. The clinical participants were older than the non-clinical participants and were more likely to be female and have CAPS-diagnosed PTSD. For traumatic events, the clinical subsample more often reported nonsexual or sexual assault, whereas the non-clinical subsample more often reported accidents, fires, or other events. The prevalence of PTSD assessed via the CAPS was 57.4% (entire sample).

**Table 2.** Characteristics of the study participants.

	Clinical subsample		Non-clinical subsample	
Variable	(*n =* 106)		(*n =* 63)	
Age, years (standard deviation)	35.87	(8.5)	20.51	(2.2)
Female, *n* (%)	106	(100)	31	(49.2)
Index trauma, *n* (%)				
Accident or fire	1	(0.9)	13	(20.6)
Natural disaster	1	(0.9)	6	(9.5)
Nonsexual assault (known assailant)	57	(53.8)	6	(9.5)
Nonsexual assault (unknown assailant)	1	(0.9)	2	(3.2)
Sexual assault (known assailant)	25	(23.6)	4	(6.3)
Sexual assault (unknown assailant)	14	(13.2)	6	(9.5)
Combat or war zone	0	(0)	0	(0)
Sexual abuse	2	(1.9)	5	(7.9)
Imprisonment	3	(2.8)	2	(3.2)
Torture	1	(0.9)	0	(0)
Life-threatening illness	1	(0.9)	1	(1.6)
Other	12	(11.3)	18	(28.6)
PTSD diagnosis, *n* (%)	94	(88.7)	3	(4.8)

Index trauma was self-rated via the Posttraumatic Diagnostic Scale and confirmed to be the same with what was obtained through the Clinician-Administered PTSD Scale (CAPS) interview. Some participants indicated that more than one event disturbed them the most; PTSD, posttraumatic stress disorder diagnosed via the CAPS. Other traumatic events included sustained domestic violence (*n =* 6), abuse (*n =* 2), sexual or power harassment (*n =* 2), sexual or nonsexual crime (*n =* 2) in the clinical subsample; bullying (*n =* 3), injury (*n =* 3), unnatural death of close person or close place (*n =* 3), painful childhood incidents (divorce, punishment) in the family (*n =* 2), victim of stalker, molester, or encounter with a stranger with a weapon (*n =* 3), other setbacks (*n =* 2), or no answer/unclear (*n =* 2) in the non-clinical subsample. Most of the non-clinical participants (87%) answered that they experienced the index trauma over a half year ago, but data for the clinical sample was not clear.

### 3.2. Descriptive statistics of re-experiencing symptoms and PTSD diagnosis

Table 1 shows the five re-experiencing symptoms of the DSM-IV (which are the same as those in the DSM-5). The number of participants who answered ‘1’ or more to each item were then separated according to whether they met the CAPS criteria for a PTSD diagnosis (PTSD+) or did not (PTSD-). Except for ‘reliving of the trauma (B3)’, the re-experiencing symptoms were experienced by over 88% of the PTSD+ participants. In particular, ‘emotionally upset when reminded of the trauma (B4)’ was experienced by the almost all of the PTSD+ participants, as well as nearly half of the PTSD- participants.

### 3.3. ROC analysis

**Figure 1.**
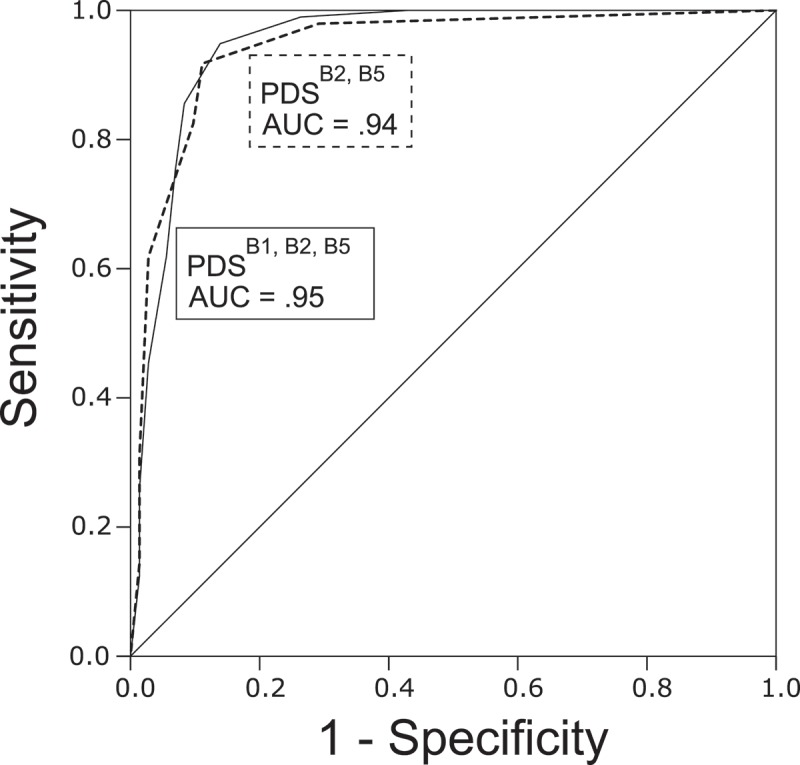
Receiver operating characteristic (ROC) curve depicting the sensitivity and specificity of the brief version of the PDS in identifying individuals with PTSD, as determined through the CAPS (DSM-IV) interview. Area under the curve (AUC) was .95 (3-item version) and .94 (2-item version). PDS, Posttraumatic Diagnostic Scale; PTSD, posttraumatic stress disorder; CAPS, Clinician-Administered PTSD Scale; DSM-IV, Diagnostic and Statistical Manual of Mental Disorders, 4th ed.

The AUCs of candidate scales are indicated in Table 3. The values were generally large (AUC = .89–.95) among all candidate scales. As indicated in Figure 1, the best ROC curve for the 3-item scales was that obtained from the candidate scale comprised ‘intrusive images (B1)’, ‘nightmares (B2)’, and ‘physiological reactions when reminded of the trauma (B5)’ (hereafter, this candidate is referred to as PDS^B1,B2,B5^). For the 2-item scales, the best ROC curve was observed for the candidate scale comprised ‘nightmares (B2)’ and ‘physiological reactions when reminded of the trauma (B5)’ (hereafter, this candidate is referred to as PDS^B2,B5^). Separate analyses for the clinical subsample (*n =* 106, including 94 PTSD), university subsample (*n =* 63, including three PTSD), and female subsample (*n =* 137, including 97 PTSD) revealed that the PDS^B1,B2,B5^ had the highest or top level AUCs among the candidate scales (Table 4).

**Table 3.** Discriminative values (95% CI), symptom severity correlation coefficients (Pearson’s *r*), and reliability coefficients (Cronbach’s alpha) of different candidates of the PDS 2- or 3-item scale applied to Japanese participants with and without PTSD.

Short versions of the PDS (DSM-IV Criteria)	Cut-point	SN	(95% CI)	SP	(95% CI)	Efficiency	(95% CI)	AUC	(95% CI)	*r*	(95% CI)	alpha
Two-item version												
(B1, B2)	1/2	94.8	(90.4–97.5)	86.1	(80.1–89.7)	91.1	(86–94.2)	0.94	(0.90–0.98)	.82	(76–86.2)	.82
(B1, B3)	1/2	90.7	(85.7–94.3)	83.3	(76.6–88.1)	87.6	(81.8–91.7)	0.91	(85.8–95.8)	.76	(68.5–81.5)	.80
(B1, B4)	2/3	88.7	(83.4–92.6)	80.6	(73.4–85.8)	85.2	(79.1–89.7)	0.90	(84.1–95.1)	.77	(69.8–82.4)	.90
(B1, B5)	1/2	97.9	(93.9–99.4)	76.4	(70.9–78.4)	88.8	(84.1–90.5)	0.93	(88.4–97.2)	.84	(78.4–87.6)	.86
(B2, B3)	0/1	94.8	(90.2–97.6)	81.9	(75.7–85.6)	89.3	(84.1–92.5)	0.92	(87.8–96.9)	.79	(72.8–84.2)	.73
(B2, B4)	2/3	86.6	(81.5–90.2)	87.5	(80.6–92.4)	87.0	(81.1–91.1)	0.93	(88.5–97.1)	.82	(75.9–86.1)	.79
(B2, B5)	**1/2**	**91.8**	**(87.1–94.8)**	**88.9**	**(82.6–93.1)**	**90.5**	**(85.2–94.1)**	**0.94**	**(90.4–98)**	.**86**	**(81.6–89.5)**	.**82**
(B3, B4)	1/2	95.9	(91.3–98.3)	76.4	(70.2–79.6)	87.6	(82.3–90.3)	0.89	(83.4–94.5)	.75	(67–80.6)	.81
(B3, B5)	1/2	93.8	(89.2–96.7)	86.1	(79.9–90)	90.5	(85.3–93.9)	0.91	(86.3–96.3)	.80	(73.7–84.8)	.82
(B4, B5)	3/4	79.4	(74.1–83)	88.9	(81.8–93.8)	83.4	(77.4–87.6)	0.91	(85.8–95.6)	.81	(74.6–85.3)	.91
Three-item version												
(B1, B2, B3)	1/2	95.9	(91.4–98.3)	81.9	(76–85.2)	89.9	(84.9–92.7)	0.94	(89.4–97.9)	.82	(76–86.2)	.85
(B1, B2, B4)	3/4	89.7	(84.7–93.2)	86.1	(79.4–90.9)	88.2	(82.4–92.2)	0.93	(88.7–97.4)	.82	(76.5–86.5)	.89
(B1, B2, B5)	**2/3**	**94.8**	**(90.4–97.5)**	**86.1**	**(80.1–89.7)**	**91.1**	**(86–94.2)**	**0.95**	**(91–98.5)**	.**86**	**(81.7–89.6)**	.**88**
(B1, B3, B4)	2/3	93.8	(89–96.9)	79.2	(72.7–83.3)	87.6	(82–91.1)	0.91	(85.6–95.8)	.78	(71–83.1)	.89
(B1, B3, B5)	2/3	92.8	(88.0–96.0)	83.3	(76.8–87.7)	88.8	(83.2–92.4)	0.93	(88.5–97.4)	.82	(76.6–86.6)	.88
(B1, B4, B5)	4/5	84.5	(79.3–88.4)	86.1	(79–91.3)	85.2	(79.1–89.6)	0.92	(87.3–96.5)	.82	(76.2–86.3)	.92
(B2, B3, B4)	2/3	94.8	(90.3–97.6)	83.3	(77.2–87)	89.9	(84.7–93.1)	0.93	(87.9–97)	.81	(75.6–85.9)	.84
(B2, B3, B5)	1/2	95.9	(91.5–98.2)	84.7	(78.9–87.9)	91.1	(86.1–93.8)	0.93	(89.1–97.6)	.85	(79.8–88.5)	.85
(B2, B4, B5)	2/3	95.9	(91.4–98.3)	80.6	(74.5–83.8)	89.3	(84.2–92.1)	0.93	(89.4–97.5)	.85	(80–88.6)	.89
(B3, B4, B5)	3/4	86.6	(81.5–90.2)	87.5	(80.6–92.4)	87.0	(81.1–91.1)	0.91	(86.2–96.1)	.80	(74.4–85.2)	.89

Bold numbers indicate values of the best candidate for the 2- or 3-item scale. PDS, Posttraumatic Diagnostic Scale; PTSD, posttraumatic stress disorder; DSM-IV, Diagnostic and Statistical Manual of Mental Disorders, 4th ed.; SN, sensitivity; SP, specificity; AUC, area under the curve.

**Table 4.** Diagnostic validity (AUC) and symptom severity correlation coefficients (Pearson’s *r*) for different candidate PDS 2- and 3-item scales applied to Japanese participants with and without PTSD, calculated separately with clinical, university, and female subsamples.

	Clinical subsample (*n =* 106)		University subsample (*n =* 63)		Female subsample (*n =* 137)	
Short versions of the PDS (DSM-IV Criteria)	AUC	*r*	AUC	*r*	AUC	*r*
Two-item version						
(B1, B2)	0.80	.58	0.91	.27	0.91	.76
(B1, B3)	0.70	.46	0.92	.29	0.92	.67
(B1, B4)	0.66	.43	0.89	.25	0.89	.70
(B1, B5)	0.69	.53	**0.91**	.**41**	**0.91**	.**78**
(B2, B3)	0.79	.59	0.81	.27	0.81	.72
(B2, B4)	0.79	.57	0.88	.26	0.88	.76
(B2, B5)*	**0.79**	.**64**	0.79	.46	0.79	.81
(B3, B4)	0.62	.41	0.88	.27	0.88	.66
(B3, B5)	0.64	.50	0.79	.44	0.79	.72
(B4, B5)	0.63	.46	0.88	.39	0.88	.73
Three-item version						
(B1, B2, B3)	0.79	.59	0.93	.29	0.90	.75
(B1, B2, B4)	0.76	.56	0.90	.26	0.89	.76
(B1, B2, B5)*	**0.78**	.**63**	**0.91**	.**40**	**0.91**	.**81**
(B1, B3, B4)	0.67	.46	0.90	.28	0.85	.70
(B1, B3, B5)	0.70	.53	0.91	.40	0.88	.75
(B1, B4, B5)	0.68	.50	0.90	.35	0.87	.75
(B2, B3, B4)	0.74	.57	0.89	.28	0.88	.75
(B2, B3, B5)	0.75	.63	0.79	.40	0.89	.79
(B2, B4, B5)	0.75	.59	0.88	.38	0.89	.79
(B3, B4, B5)	0.64	.48	0.88	.38	0.85	.73

* indicate values of the best candidate for the 2- or 3-item scale in the whole sample analysis. Bold numbers indicate the best candidates in the sub-sample analysis. AUC, area under the curve.

The optimal cut-off scores were a total score of ≥3 and ≥2 for the PDS^B1,B2,B5^ and the PDS^B2,B5^, respectively. At these cut-off scores, the sensitivity, specificity, and efficiency of the scales were calculated. These values for the PDS^B1,B2,B5^ and PDS^B2,B5^ were generally high (>85%). The PDS^B1,B2,B5^ showed better sensitivity (94.8%) than did the PDS^B2,B5^ (91.8%), but the PDS^B2,B5^ had a somewhat better specificity (88.9%) than did the PDS^B1,B2,B5^ (86.1%). The efficiency value and the AUC indicated that the general diagnostic validity for these two scales was almost the same, although it was slightly higher for the PDS^B1,B2,B5^ than for the PDS^B2,B5^. The positive and negative predictive values are not shown in Table 3, as these were originally valuable indices when the sample has a ‘representative’ respondent pool; this was not the case for our sample. Nevertheless, the positive and negative predictive values were both high for the PDS^B2,B5^ (91.8% and 88.9%, respectively) and PDS^B1,B2,B5^ (90.2% and 92.5%, respectively).

### 3.4. Correlational analysis

As shown in Table 3, there were generally strong positive correlations between the total scores of the candidate scales and the CAPS severity score (Pearson’s *r *= .75–86). The strongest correlations among the 2- and 3-item candidate scales were for the PDS^B2,B5^ (*r *= .86, *p* < .01) and the PDS^B1,B2,B5^ (*r *= .86, *p* < .01), respectively. The PDS^B2,B5^ and the PDS^B1,B2,B5^ also demonstrated the highest values for the subsample analyses (Table 4).

### 3.5. Reliability analysis

As shown in Table 3, there were generally sufficient internal consistency coefficients for the candidate scales (Cronbach’s alpha = .73–.92). The internal consistency was high for the PDS^B1,B2,B5^ (alpha = .88) and for the PDS^B2,B5^ (alpha = .82).

## 4. Discussion

Here, we sought to develop a short self-rating scale for screening PTSD, which focused on re-experiencing symptoms. Before discussing the obtained values, it is important to note our sample. Generally, in examining scale validity, researchers should ensure that the sample includes individuals with a range from mild to severe symptoms. In contrast, our examination was based on the data of a previous study, combining Japanese female outpatients suffering from traumatic experiences and university students who had subjective traumatic experiences. In short, our sample might have included only those with mild or severe symptoms, with few in the middle of the spectrum, thus resulting in bias. Therefore, it is important to note that the values obtained in the present study may be overestimated due to our ease of being able to discriminate between PTSD+/–. Nevertheless, despite this potential limitation, our original interest was in clarifying which symptoms (B1–B5) contribute more to a discrimination of PTSD +/-, assuming the possibility of overestimated values, regardless of the candidate scale(s) we employed. Therefore, the present results are useful as preliminary evidence toward developing a new short, PTSD scale.

The present results showed that the 3-item scale comprising ‘intrusive images (B1)’, ‘nightmares (B2)’, and ‘physiological reactions when reminded of the trauma (B5)’, and the 2-item scale of ‘nightmares (B2)’ and ‘physiological reactions when reminded of the trauma (B5)’ had the highest AUCs. Their correlations with PTSD severity score were at least .86 and internal consistencies (alpha) were ≥ .82. Notably, the observed values were generally higher than were those for previous short scales. In particular, compared with the similar 2-item re-experiencing scales based on the PCL, the PDS^B2,B5^, and PDS^B1,B2,B5^ had somewhat lower but similar (Lang & Stein, 2005; Study 2) or higher (Tiet et al., 2013; Sample 2) sensitivity and obviously higher specificity (Lang & Stein, 2005; Tiet et al., 2013). Additionally, in comparison with the PC-PTSD (Prins et al., 2003), which is a 4-item scale widely used in primary care settings, the PDS^B2,B5^ and PDS^B1,B2,B5^ showed greater sensitivity despite fewer items, but similar specificity.

These results can be explained by the following three reasons. First, our procedure for choosing items was systematic: we created a pool of candidate scales and chose the most appropriate ones in relation to a CAPS diagnosis (rather than PCL-evaluated severity). Second, our new scales were created from items in the PDS, and used a 4-point rating scale (with total ranges of 0–6 and 0–9 for the 2- and 3-item scales, respectively). In contrast, the PC-PTSD uses a binary rating scale (yes/no; total scores range of 0–4); as such, the PTSD classification might be more precise in our scales because of the more detailed scoring procedure. Third, it might be easy to discriminate PTSD +/- in our sample. Our clinical subsample comprised people who had visited psychiatric clinics; thus, they might present with more severe symptoms, be less hesitant to confess their own symptoms, or express their own symptoms more explicitly to obtain others’ support when compared with the non-clinical subsample. Previous studies might have found it more difficult to discriminate PTSD +/- because their samples reflected real clinical populations and included more people with subtle symptoms.

Although our scales should not be immediately applied to a clinical population, the chosen re-experiencing scales had adequate validity and generality. When examined by the specific re-experiencing items chosen, B1 was selected for both our scale and the 2-item version of the PCL (Lang & Stein, 2005). The B1 was rated as ‘1’ or more by almost all of the PTSD+ participants in the present study (97%), and the B1 was also frequently experienced by PTSD+ participants in the original PDS validation study (98%; Foa et al., 1997); thus, this symptom is likely common among those with PTSD and is actually useful for PTSD screening. With respect to B2, fewer PTSD+ people reported this symptom in the present study (89%) and the original PDS validation study (78%; Foa et al., 1997). This item was not chosen for the 2-item version of the PCL but was chosen in this study. The reason for the difference could be that B2 was little experienced by the PTSD- group (8% and 32%, in the present study and Foa et al., 1997, respectively), and, despite being helpful for discriminating PTSD+/-, was less helpful for representing total PCL-evaluated severity. B3 was chosen neither for our scale nor for the 2-item version of the PCL (Lang & Stein, 2005). B3 was the least-experienced symptom of the five re-experiencing symptoms in the PTSD+ group (79%), and a lower tendency for PTSD+ to report B3 (74%) was also reported by Foa et al. (1997). B3 might not affect the sum score of the 2- or 3-item scale, and thus may not contribute to discriminating PTSD+/-. In contrast, B4 was the most commonly reported symptom by PTSD+ (99%) and was chosen for the 2-item version of the PCL; however, it was not chosen for our scale. It was also experienced by nearly half of the PTSD- participants (49%), so it might not be helpful for discriminating PTSD+/-. In Foa et al. (1997), B4 was the most experienced symptom by the PTSD- (73%). Finally, B5 was chosen for our scale but not for the 2-item version of the PCL (Lang & Stein, 2005). B5 was experienced by many PTSD+ participants (97%) but fewer PTSD- participants (26%), suggesting that it might be useful for diagnosing PTSD. In summary, B1, B2, and B5 were experienced by about 90% or more PTSD+ participants but by 35% or fewer PTSD- participants.

With respect to the length of the scale, our results indicated similar values between 2- and 3-item versions. Although higher values tended to be observed for the 3- rather than 2-item version, the 95% confidence intervals of 3-item version included the corresponding values of 2-item version. There is currently no definitive evidence by which to recommend either the 2- or 3-item version. Interestingly, B2 and B5 were common to the 3- and 2-item versions. Both relate to physiological responses; thus, physiological assessments might be promising methods of screening for PTSD in the future.

Several limitations to this study must be mentioned. First, as noted above, the study sample did not include an appropriate spectrum of people with mild to severe PTSD symptoms, and the results were based on separate subsamples. The analyses with the clinical and university subsamples (Table 4) revealed stability in the results for the 3-item scale but not the 2-item scale. Therefore, validation of this instrument in a larger population (e.g. in an evacuation situation) should be performed in the future study. Second, most of the PTSD+ participants were female patients with interpersonal violence trauma. Although results with the whole sample did not differ from results for the female subsample, at least for the 3-item scale (Table 3, 4), gender differences in trauma types, psychological and biological responses, and subsequent outcomes have been suggested (Olff, Langeland, Draijer, & Gersons, 2007), and it is uncertain whether B1, B2, and B5 would be essential elements for a PTSD diagnosis in men. Nevertheless, even if our findings might be specific to a female sample, the present study has clinical value, because women have a higher risk of developing PTSD than men (Brewin, Andrews, & Valentine, 2000). Third, our results were based on *a posteriori* selection of response data from the original full-length PDS answer dataset, so ‘framing’ or ‘carryover’ effects of prior questions might be included. Whether the PDS^B1,B2,B5^ and PDS^B2,B5^ would show the same psychometric characteristics when presented by themselves should be considered in a future study.

## 5. Conclusion

For identifying high-risk individuals with PTSD during an evacuation situation, two items (‘nightmares (B2)’ and ‘physiological reactions when reminded of the trauma (B5)’) or three items (‘intrusive images (B1)’, ‘nightmares (B2)’, and ‘physiological reactions when reminded of the trauma (B5)’) provide adequate validity based on ROC analyses and when compared to the CAPS as an external criterion. Therefore, the present study represents an important step towards the development of a new short PTSD screening scale. We expect that further validation of the scales among a wide range of individuals who have experienced various traumas will help provide a higher quality screening tool for PTSD, particularly for evacuation scenarios and will improve the efficiency of early intervention.

## Supplementary Material

Supplementary material

## Note

1. We also adopted item response theory (IRT) to our data to understand characteristics of each re-experiencing item. We confirmed that the set of PDS 22, 23, and 26 (3-item version chosen by the ROC analyses) or PDS 23 and 26 (2-item version chosen by the ROC analyses), thought to offer a certain level of information for low to high latent PTSD, served the purpose of screening a wide range of people. For more information, please contact Yoshiharu Kim (kimpds3@gmail.com).
