# Supplementary material for: A new short version of the Posttraumatic Diagnostic Scale: validity among Japanese adults with and without PTSD
Source: Eur J Psychotraumatol. 2017 Sep 5;8(1):1364119. doi: 10.1080/20008198.2017.1364119 (PMC5614216; doi:10.1080/20008198.2017.1364119)
Supplement: Supplementary material [file ZEPT_A_1364119_SM3805.docx]

# Supplementary material for review


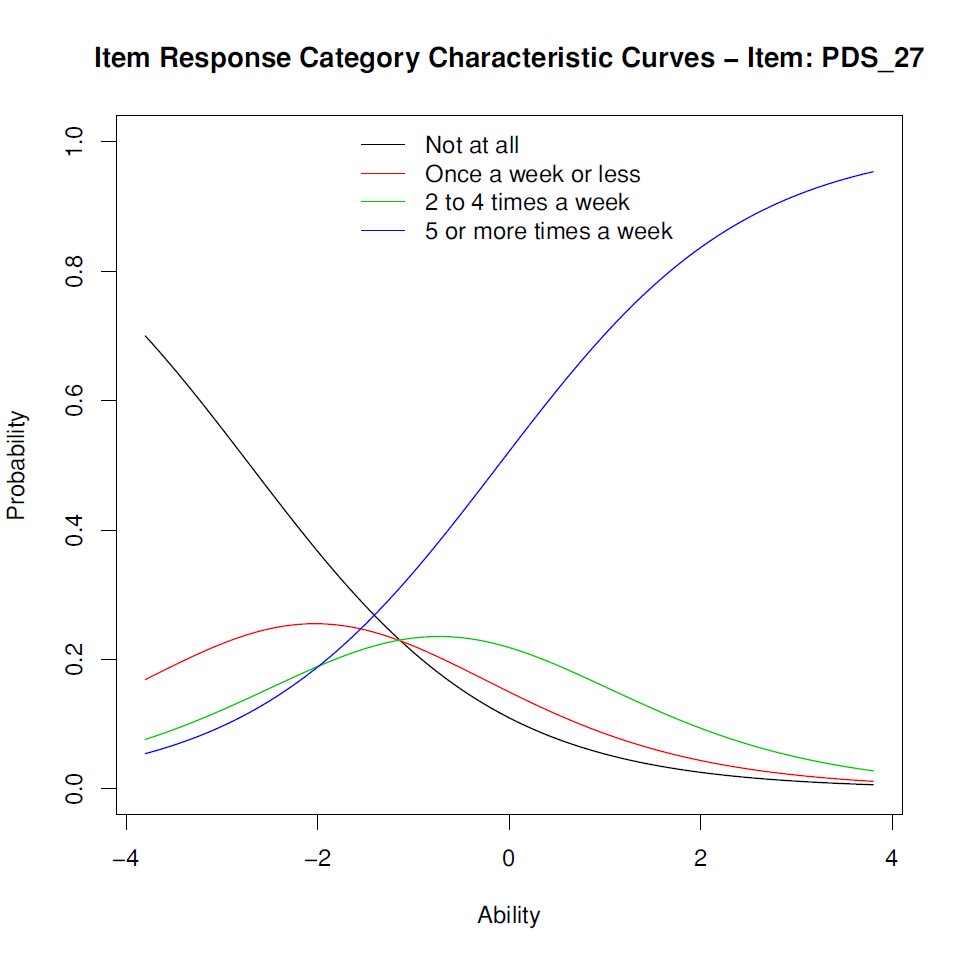

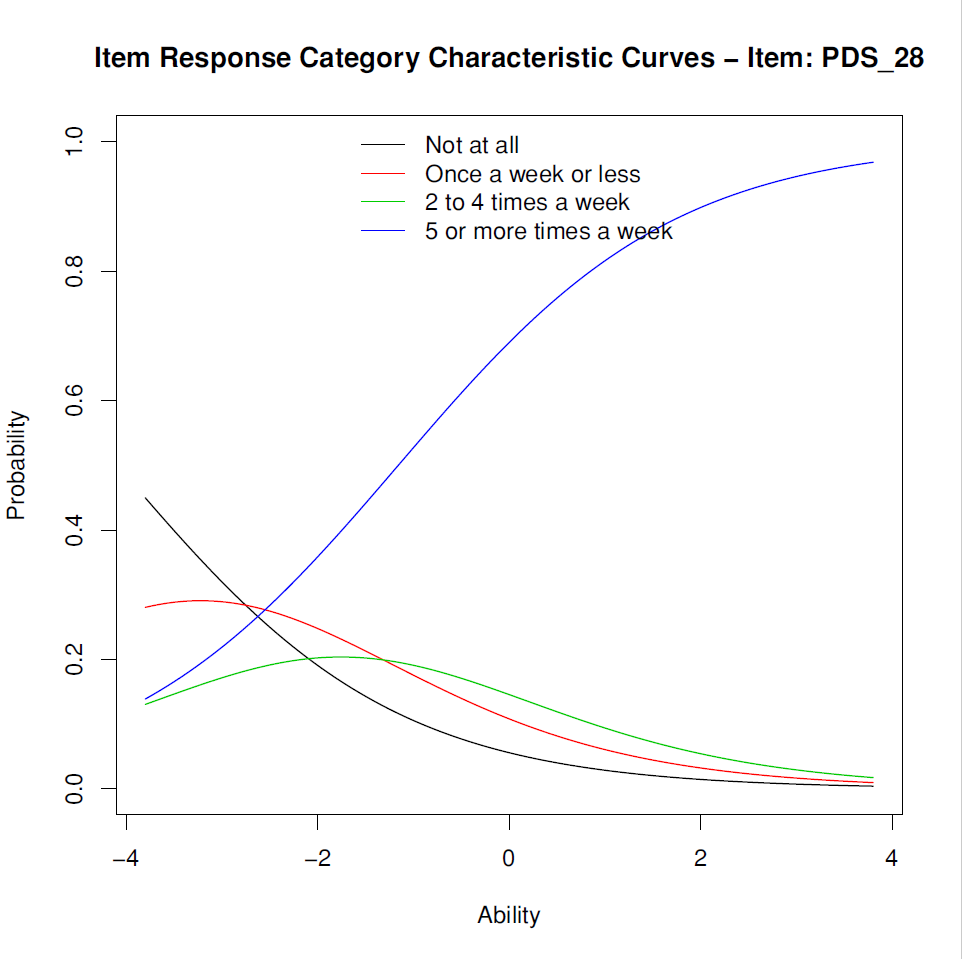

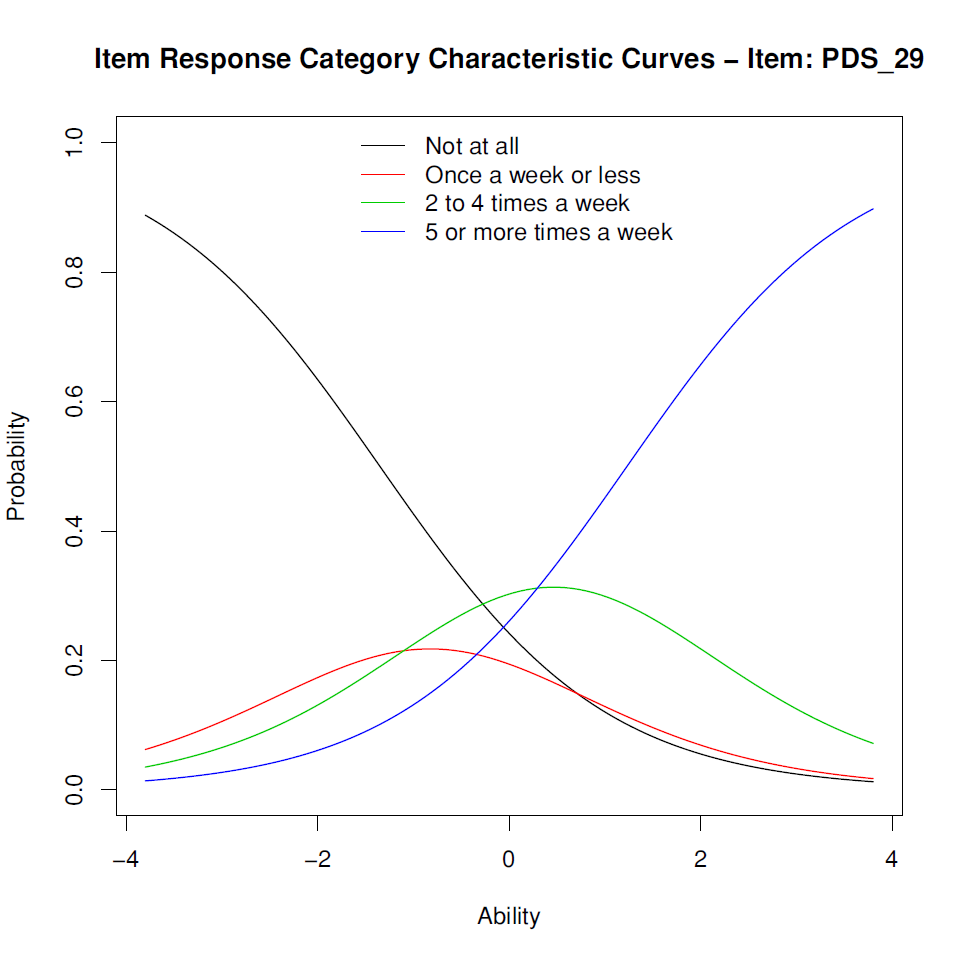

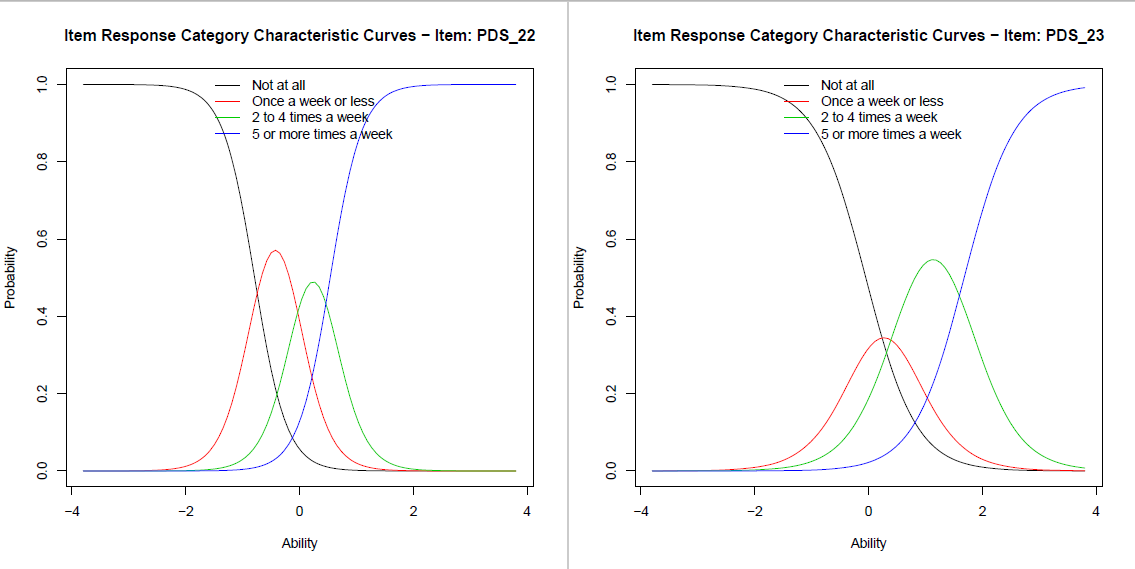


Figure 1. Item response category characteristic curves for the avoidance items of PDS 27 (top left), 28 (top right), 29 (bottom left), and re-experiencing item 22 (bottom right, as a reference). PDS 27, ‘trying to avoid thoughts or feelings related to the trauma’; PDS 28, ‘trying to avoid activities, situations, or places that remind you of the trauma’; PDS 29, ‘not being able to remember important parts of the trauma’; PDS 22, ‘intrusive images’.

Figure 2. Item response category characteristic curves for PDS 22, 23 (top row), 24, 25 (middle row), and 26 (left bottom), as well as item information curves for the 5 re-experiencing items (right bottom). PDS 22, ‘intrusive images’; PDS 23, ‘nightmares’; PDS 24, ‘reliving of the trauma’; PDS 25, ‘emotionally upset when reminded of the trauma’; PDS 26, ‘physiological reactions when reminded of the trauma’.


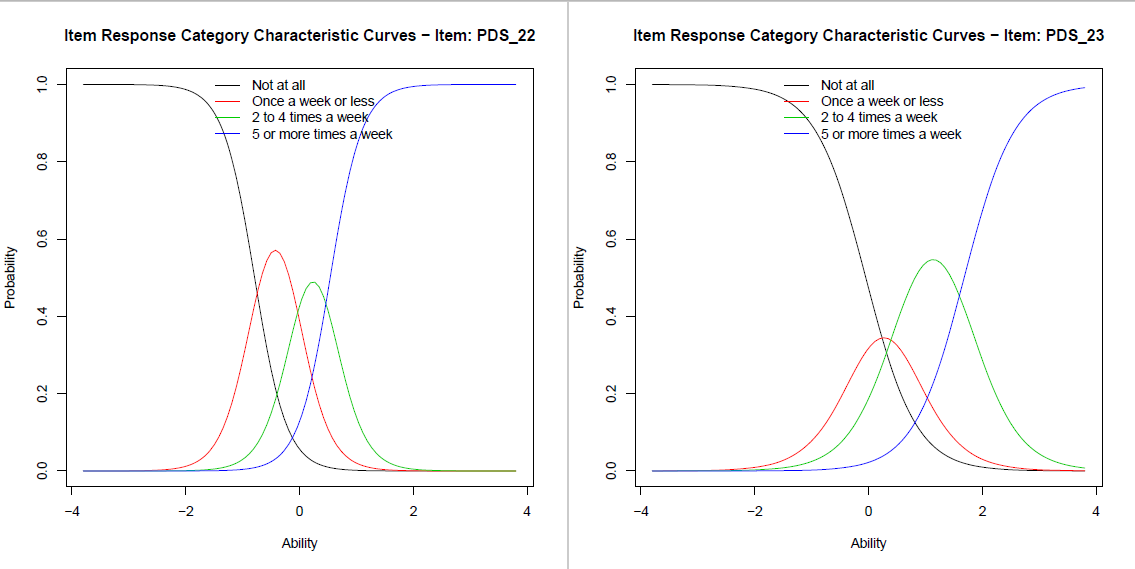

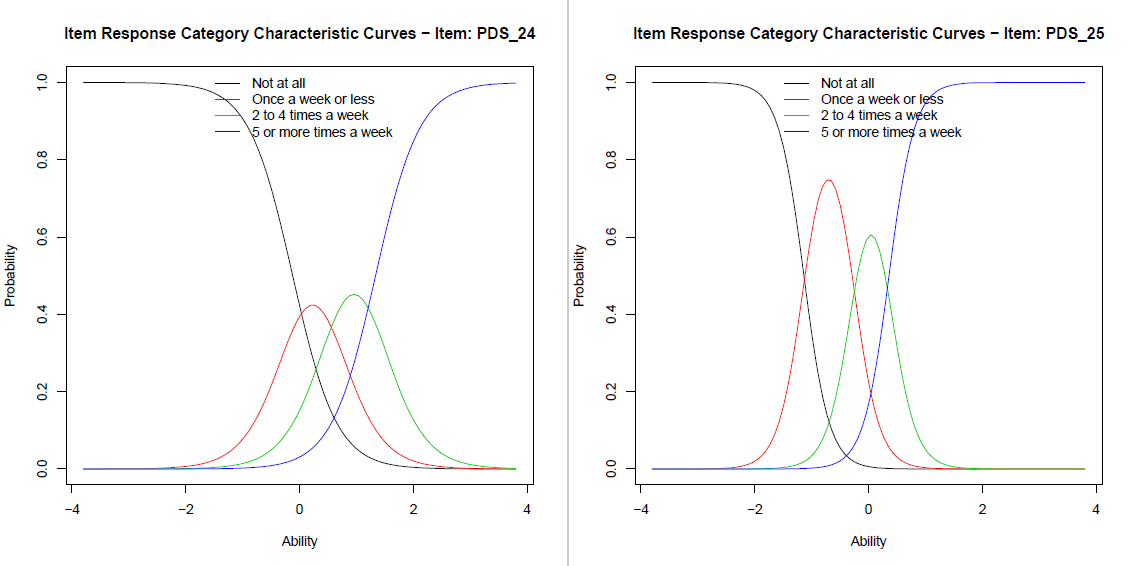

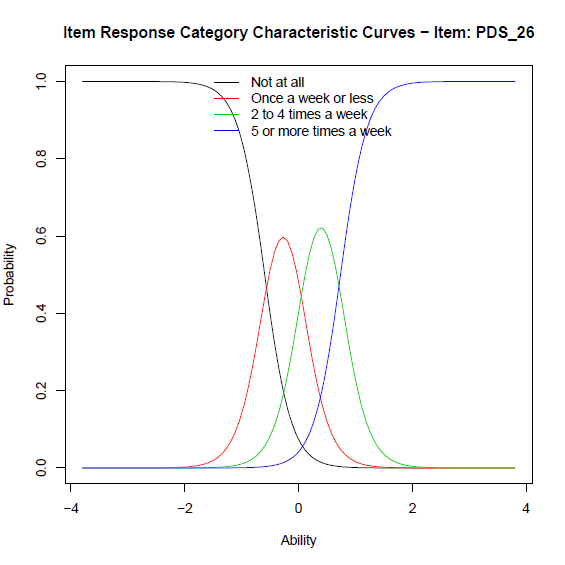

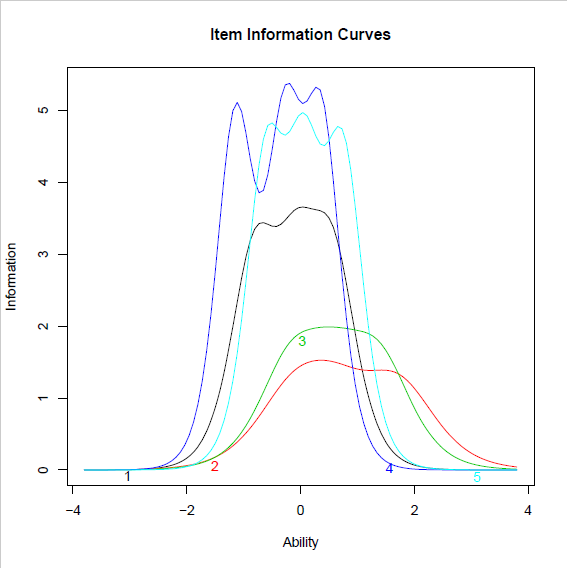


PDS 22

PDS 23

PDS 24

PDS 25

PDS 26
